# Portable Real-Time Detection of Pb(II) Using a CMOS MEMS-Based Nanomechanical Sensing Array Modified with PEDOT:PSS

**DOI:** 10.3390/nano10122454

**Published:** 2020-12-08

**Authors:** Yi-Kuang Yen, Chao-Yu Lai

**Affiliations:** 1 Department of Mechanical Engineering, National Taipei University of Technology, Taipei 106, Taiwan; t106568056@ntut.edu.tw; 2 Institute of Mechatronic Engineering, National Taipei University of Technology, Taipei 106, Taiwan

**Keywords:** CMOS MEMS, nanomechanical sensor, conductive polymer, lead ion detection

## Abstract

Detecting the concentration of Pb^2+^ ions is important for monitoring the quality of water due to it can become a health threat as being in certain level. In this study, we report a nanomechanical Pb^2+^ sensor by employing the complementary metal-oxide-semiconductor microelectromechanical system (CMOS MEMS)-based piezoresistive microcantilevers coated with PEDOT:PSS sensing layers. Upon reaction with Pb^2+^, the PEDOT:PSS layer was oxidized which induced the surface stress change resulted in a subsequent bending of the microcantilever with the signal response of relative resistance change. This sensing platform has the advantages of being mass-produced, miniaturized, and portable. The sensor exhibited its sensitivity to Pb^2+^ concentrations in a linear range of 0.01–1000 ppm, and the limit of detection was 5 ppb. Moreover, the sensor showed the specificity to Pb^2+^, required a small sample volume and was easy to operate. Therefore, the proposed analytical method described here may be a sensitive, cost-effective and portable sensing tool for on-site water quality measurement and pollution detection.

## 1. Introduction

Global water resources have been profoundly affected by human activities in the past few years, and a significant number of places in the world are facing serious problems of water supply and drinking water quality. In many places in the world the concentration of heavy metals (HM) in drinking water is higher than the international standard value. Millions of people are currently known to have chronic heavy metal poisoning; this has become a global public health problem, and 1.6 million children die each year from diseases caused by contaminated drinking water [1]. Among these heavy ions, lead ion (Pb^2+^) is known to be one of the most poisonous ones with an increasing negative potential impact on global human health [2]. Excessive intake of even low-level lead ions can damage the central nervous system and peripheral. For infants and children, excessive intake is mainly manifested in physical or mental developmental delay. For adults, it is mainly reflected in kidney problems and high blood pressure. For pregnant women, the consequences are more serious, possibly including hypertension and nervous system developmental delay, with potential neurobehavioral and intellectual development after birth [3,4]. According to the World Health Organization (WHO) [2,5], and the US National Environmental Protection Agency (EPA) [6], the critical concentration of lead ions in natural waters is reported as 0.05 ppm (0.24 mM) and 0.015 ppm (72.4 nM), respectively. Consequently, the demand for developing lead ion sensors for aqueous detection to ensure healthy drinking water in urban and rural areas is rapidly increasing.

At present, several methods are used for the detection and analysis of heavy metal ions, including atomic absorption spectrometry (ASS) [7], inductively coupled plasma optical emission spectrometry (ICP-OES) [8], mass spectrometry ICP-MS [9], high-performance liquid chromatography (HPLC) [10], electrochemical sensors [11,12,13,14], colorimetric sensors [15,16], optical-based sensors [17], fluorescence sensors [18] and the enzymatic inhibition method [19]. Although these analytical methods have the advantages of high sensitivity, good selectivity and high accuracy, the signal acquisition equipment is expensive and bulky. Moreover, the detection process is cumbersome, requiring a large amount of sample which needs time-consuming preparation and pre-purification. These methods are also unsuitable for immediate or continuous on-site environmental monitoring. Recently, a black phosphorus functionalized optical fiber sensor based on a microfiber coil resonator was fabricated for Pb^2+^ detection [20]. Although the sensor had advantages, including a simple structure, ease of fabrication, low cost, low loss and interrogation simplicity, it did not achieve to be miniaturized and portable. Another lately work demonstrated the colorimetric sensing of Pb^2+^ ion based on metal induced aggregation of N-decanoyltromethamine (NDTM) capped gold nanoparticles (AuNPs) [21]. The method was quite simple, rapid, cost-effective and did not require sophisticated instrumentation, but the amount of tested samples were larger (1 mL) and the measurement system (spectrophotometry) was enormous. Therefore, the development of sensing technology with the characteristics of fast detection, low cost, portability and simple operation may be regarded as providing suitable methods for on-site monitoring of water pollution and poisonous substances.

Thanks to the development of micromachining technology in the past decade, several microdevices may have features to allow them to be a potential alternative to the current detection methods, such as low volume of samples, high degree of system integration, automation of measurement, easy handling and short response time [22,23]. Taking advantages of those microfabrication and integration advances, many new micromachined based analytical methods have recently been proposed to detect lead ions [24,25,26,27]. Teh et al. developed a DNAzyme-based quartz crystal microbalance (QCM-D) sensor for the detection of lead ions [24]. They used oligonucleotide functionalized gold nanoparticles for frequency and dissipation amplification to enhance the performance of the sensor; the lead ion concentration can be quantified in real time and the sensor detection limit was 14 nM for lead ions, with excellent selectivity. Since the frequency change of the QCM sensor was the overall response to the mass absorption of ionic liquid and the resulting viscosity change in room temperature, the zero shift of the fundamental frequency of the resonance may make small signals unobservable [28]. Zhou et al. reported a field-effect transistor (FET) device-based sensor utilizing reduced graphene oxide/L-glutathione reduced (rGO/GSH)-AuNP hybrid structure for detecting Pb^2+^ ions in an aqueous environment. The method showed a very fast response of Pb^2+^ detection (1–2 s) and the sensitivity of 10 nM with the selectivity against other metal ions [25]. Peng et al. developed a microcantilever-based Pb^2+^ sensor modified with a DNAzyme molecule. This microcantilever sensor could detect Pb^2+^ sensitively and selectively in an aqueous solution. This sensor could be regenerated by using the Pb^2+^ chelator; the detection of limit was 10 nM with good selectivity, but the deflection signals were measured by using an optical readout system which may not be suitable for portable application [26]. Although these sensing devices can achieve miniaturization, high sensitivity and fast response, the fabrication processes of these devices were difficult to standardize, resulting in device instability and high manufacturing cost.

In this study, we aimed to develop a portable nanomechanical sensing platform for detecting lead ions in aqueous samples as verification of the feasibility and sensitivity of the sensor. We adopted the commercial standard CMOS process and the MEMS post-process (TSMC 0.35 µm 2P4M CMOS MEMS process) to fabricate sensing chips. Meanwhile, we designed the on-chip thermal effect self-elimination configuration [29] integrated into the CMOS process for rendering the whole sensing system portable. In addition, we modified the poly (3,4-ethylenedioxythiophene):poly(styrenesulfonate) (PEDOT:PSS) on the sensing microcantilever by using a direct ink extrusion printing. The coating process also can be standardized by integrating three-dimensional motion control module. Owing to the advantages of the standard process, the sensing devices can achieve a high yield rate (>90%), small device variance, high compatibility with integrated circuits and mass production for reducing the cost. Furthermore, not much work has been reported for studying the surface stress change of the CMOS MEMS-based microcantilever which was induced by the interaction of PEDOT:PSS film and lead ions [30,31,32]. The package of the device was compact and lightweight as well as the sample volume of each measurement only needed 5 μL and required no pretreatment of samples. In short, the proposed sensor has the potential to be a portable real-time lead ion detection tool with quantitative analysis, low-cost production and no requirement of operational professionals.

## 2. Materials and Methods

### 2.1. Design and Fabrication of Microcantilever Sensing Array Device

**Figure 1:**
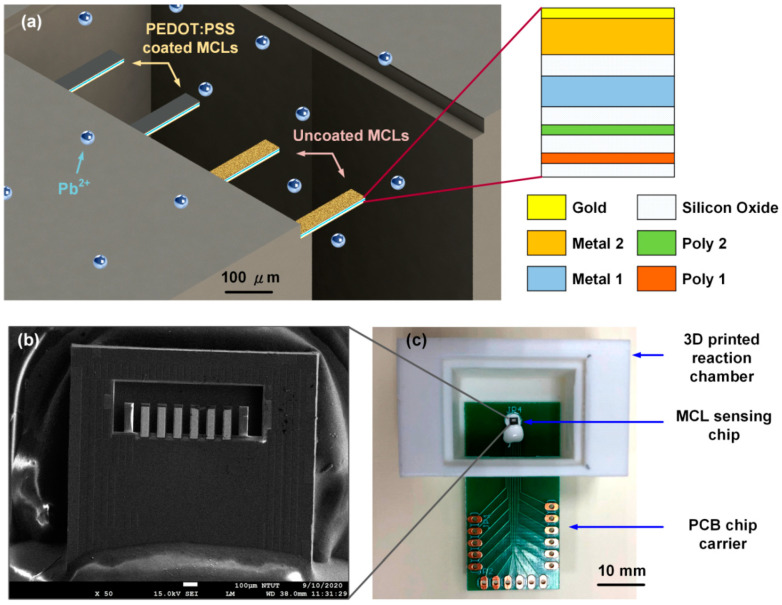
(**a**) schematic diagram of the detecting concept and multilayer structure of CMOS MEMS-based microcantilever sensing array; (**b**) the SEM photograph of the microcantilever sensing array chip wire bonded on the printed circuit board (PCB) chip carrier; and (**c**) the portable electric readout package of the microcantilever sensing array for Pb^2+^ detection.

The microcantilever sensing array chip was designed by using the Laker^TM^ software provided by Taiwan Semiconductor Research Institute (TSRI). After the chip design was completed, it was verified through the design rules of the standard manufacturing process to avoid unpredictable errors in chip production. The chip size is 1.5 mm^2^. The schematic diagram of the microcantilever sensing array for Pb^2+^ detection is shown in Figure 1a. The chip layout includes three parts: The sensing microcantilever array, embedded metal temperature sensor and fixed piezoresistors. The sensing microcantilever was designed as a four-layer structure (2P2M) in which the polysilicon layer (Poly2) served as the piezoresistive layer for signal inducing. The other layer, Poly1, was used to protect the piezoresistive layer and serve as the structural layer. The main structural layers were Metal1 and Metal2. The size of the microcantilever was designed to be 200 μm in length, 43 μm in width and 3.5 μm in thickness. The resistance of the piezoresistive microcantilever was calculated to be about 7.2–8.1 kΩ according to the parameter data provided by TSRI. To avoid the sensor signal being affected by environmental temperature changes, the thermal effect self-elimination scheme (described in the Supplementary Materials S1) was integrated on the sensing chip. The aluminum Metal1 layer served as the temperature wire to monitor the on-chip temperature for the thermal compensation purpose. The line width of the aluminum wire was 1 μm and the length was about 2100 μm, whereas the resistance value of the metal wire was 150 Ω.

After the confirmation according to the design rule, the sensor was fabricated through the TSMC 0.35 µm 2P4M CMOS MEMS process. In the post-processing process, the silicon oxide was removed by anisotropic dry etching to define the shape of microcantilevers, followed by a thin gold layer being deposited on the Metal 2 layer for the further surface modification. Finally, microcantilevers were released by using the isotropic dry etching process. The SEM image of microcantilever sensing array chip is shown in Figure 1b. The chip was further wire bonded on the designed printed circuit board (PCB) for the electric readout (Figure 1c). The size of the PCB was 40 mm by 20 mm and the contact was bare copper without tin spraying treatment, to prevent the tin from making the aluminum wire unable to firmly bond to the electrode contact. For the sensing chip to work in a liquid environment, the silicone glue (DOW CORNING^®^ SE4486) was applied to protect the bonding wire and electrode and cured in the air for 24 h. For the sensor to achieve portable and replaceable usage, the 3D printed chamber was designed and made (Figure 1c). The chamber has a movable upper cover so that the sample solution can be easily added, while also protecting the sensor from the interference of external substances during the measurement.

### 2.2. Experimental Chemicals and Instruments

PEDOT:PSS 1.3 wt % dispersion in H_2_O (483095), Pb(NO_3_)_2_ powder (1.07398), 1000 mg/L standard solutions Ca(NO_3_)_2_ (1.19778), KNO_3_ (1.70230), Cr(NO_3_)_3_ (1.19779), Mn(NO_3_)_2_ (1.19789), Zn(NO_3_)_2_ (1.19806) and 99.8% ethanol solutions were all purchased from Sigma Aldrich, Taipei, Taiwan. Sodium chloride was bought from First Chemical Manufacture Co., Taipei, Taiwan. Millipore Milli-Q water of resistivity 18.2 MW cm (at 25 °C) was used in all aqueous solutions. In this work, the pH value of all the sample solutions was 7.0, since high pH (pH > 10) affects the PEDOT:PSS sensing film of the sensor [33]. In real applications, the test samples would be subjected to a preliminary simple pH test and then adjusted pH value to be approximately 7 for the measurement.

An optical microscope (Nikon LV-150, Tokyo, Japan) and a scanning electron microscope (FE-SEM, JSM-7610F, JEOL, Tokyo, Japan) were used to characterize the microcantilever sensor array chip, respectively. A three-axis micropositioner (Sadhudesign Co., Taipei, Taiwan) was employed for the direct ink extrusion coating of a conductive polymer onto the microcantilever surface. A simple microscope with a real-time CMOS camera capture system was utilized for inspecting the coating processes. The temperature control platform (Honghui Optoelectronics 10TEC-150, Unice E-O Services Inc., Taoyuan, Taiwan) was used to calibrate the embedded on-chip temperature sensor for the thermal effect self-elimination method. The multi-function digital electric meter (NI PXI-4071, National Instruments, Taipei, Taiwan) with the divider and the relay matrix (NI PXI-2503, National Instruments, Taipei, Taiwan) were integrated for electrical measurements.

### 2.3. Sensor Surface Modification

PEDOT:PSS was utilized as the recognition layer of lead ions in expectation of inducing the surface stress change of the microcantilever. The sensor chip was cleansed by ethanol for 30 s. A 1.3 wt % PEDOT:PSS ink was coated onto the surface of the microcantilever by using the direct ink extrusion printing method. The schematic plot of PEDOT:PSS coating process was shown in Figure S2. The ink was pumped at a flow rate of 0.02 mL/h through the tubing to the glass microneedle with an outer diameter of 40 μm. The glass microneedle was clamped onto a three-axis linear stage which controlled the coating direction of the PEDOT:PSS ink. The coated sensor chip was then dried on the hotplate at 85° C for 10 min to form a film on the microcantilever surface. The coated dry film thickness can be controlled and calculated from the pump flow rate, solid content of the ink, the coating speed and the coating width. The relation that determines the film thickness was described in Supplementary Materials Figure S2. As result, the thickness can be calculated as 1.8 μm.

## 3. Results and Discussion

### 3.1. Sensor Characterizations

The gauge factor of the CMOS MEMS-based microcantilever sensor can be calculated by measuring the endpoint displacement (Δz) and the resistance value change (ΔR) together with the known dimension of the microcantilever. According to the experimental results (described in the Supplementary Materials Figure S3), ΔR/Δz = 5.4483, and the initial resistance value of the micro-cantilever at 25 °C is R0 = 7.23 kΩ. From the material parameters of the microcantilever structure, the position of the neutral axis obtained was 1638 nm counted from the bottom and the distance of the piezoresistive layer from the neutral axis of the microcantilever was 946 nm. Therefore, the gauge factor of microcantilever sensor obtained was 13.

**Figure 2:**
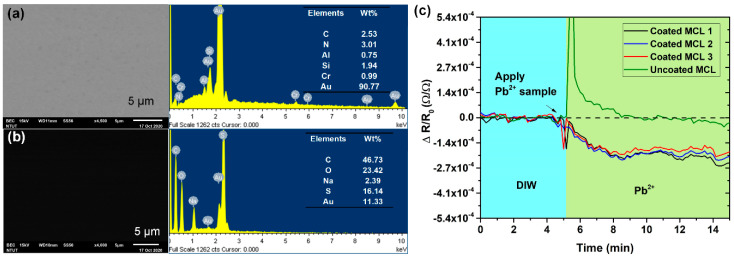
(**a**) the SEM photograph and EDS spectrum of the gold surface of microcantilever; (**b**) the SEM photograph and EDS spectrum measured from the poly (3,4-ethylenedioxythiophene):poly(styrenesulfonate) (PEDOT:PSS) coating film on the surface of microcantilever; and (**c**) response in resistance change as a function of time, for microcantilevers coated with and without the PEDOT:PSS sensing film on the gold surface after the injection of a 100 ppm Pb^2+^ solution.

FE-SEM was implemented to analyze the coated material on the surface of the microcantilever. Figure 2a,b) express the SEM picture and EDS spectrum of the gold coated surface and modified PEDOT:PSS surface of the microcantilever, respectively. From EDS spectrums, two sample surface compositions were compared. From the right-hand side of Figure 2b, the PEDOT:PSS modified surface exhibits the presence of sulfur (S) content (wt % 16.14) in comparison with the gold coated surface, which indicates the presence of the PEDOT:PSS layer. Moreover, the nanomechanical sensing method itself can be used as a tool for the thin film stress study [34,35]. In order to verify whether the PEDOT:PSS coated layer reacted with Pb^2+^, the sensor array with coated and uncoated microcantilevers was examined in the Pb^2+^ sample solution. The sensor array first had 5 μL of deionized water added and was measured for two min. After that, the deionized water was sucked out and 5 μL of 100 ppm Pb^2+^ sample solution was added for a further 5 min of measurement. As shown in Figure 2c, the microcantilevers were pre-equilibrated in deionized water before applying the Pb^2+^ solution. Moreover, it can be found that only the PEDOT:PSS functionalized microcantilevers had reaction signals with resistance change of −1.347 Ω on average, and the unmodified microcantilever had no reaction to lead ions relative to the deionized water. This response in resistance changes presents the microcantilever bending due to the change of induced surface stress resulting from the PEDOT:PSS oxidized by lead ions [31,36].

### 3.2. Detection of Lead Ions

**Figure 3:**
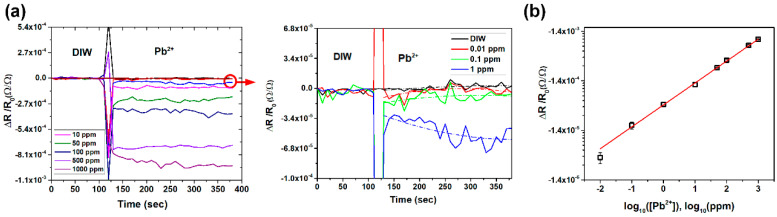
(**a**) Signal responses of PEDOT:PSS-modified CMOS MEMS-based nanomechanical sensors in solutions with different concentrations of lead ions and (**b**) the analysis of the linear relationship between the logarithmic signal response of the sensor and the logarithmic concentration of lead ions.

As illustrated in Figure 3a, the responses in relative resistance changed as a function of time when the microcantilever sensing arrays were exposed to various Pb^2+^ concentrations from 0.01 and 1000 ppm. As the Pb^2+^ concentration continued to increase in the sample solutions, the signal response of relative resistance accordingly changed by increasing negatively compared to the signal of the deionized water. These signal responses indicated the microcantilever bending down due to the induced surface stress from the oxidation of the PEDOT:PSS. It was also found that the response time of the sensor to Pb^2+^ ions was within 1 min; however, the measuring time in this study still operated for 5 min in order to obtain the steady state signals. The fluctuation of signals during measurements could be observed. The sensing platform was placed on a table without vibration-proof apparatus, to simulate a portable testing environment. Although the noise of mechanical vibration cannot be neglected, the signals corresponding to low Pb^2+^ concentration could still be recognized by trend line fitting. The thermal effect elimination method was successfully applied so that the sensing platform could perform without an additional temperature control system.

Figure 3b shows the calibration curve of the relative change in resistance upon exposure of the sensor to Pb^2+^ solutions with concentrations ranging from 0.01 to 1000 ppm. The coefficient of variation induced by different sensors was found to be within 8%. The data showed reasonably good reproducibility from each sensing chip to chip. The logarithmic signal response showed a linear relationship with the logarithmic concentration of Pb^2+^ (each concentration measured by using a least three sensors, n≥3) at the measuring time of 5 min. The sensitivity of the sensor can be expressed as:

(1) $$\log_{10}[-\Delta R/R_{0}]=0.445\log_{10}[{\mathrm{Pb}}^{2+}\;\mathrm{conc}.]-4.359,\;0.01\;\mathrm{ppm}\leq [{\mathrm{Pb}}^{2+}\;\mathrm{conc}.]\leq 1000\;\mathrm{ppm}$$

where the coefficient of determination is 0.99. The limit of detection (LOD) is defined as triple the value of the signal response in a blank solution (Δ*R*/*R*_0_ ≅ −1.41 × 10^−6^). The LOD of the CMOS MEMS-based nanomechanical sensor of Pb^2+^ detection is 0.005 ppm (Δ*R*/*R*_0_ ≅ −4.23 × 10^−6^).

### 3.3. Selectivity

**Figure 4:**
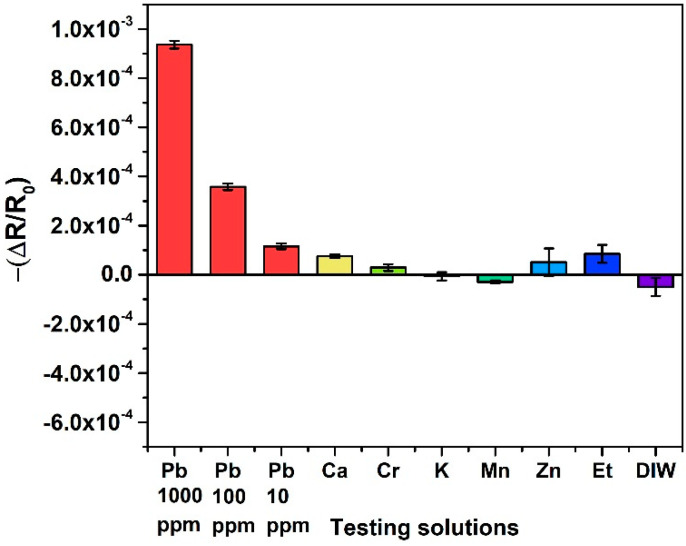
Sensor responses in relative resistance change to the target Pb^2+^ solutions (1000 ppm; 100 ppm; 10 ppm of Pb(NO_3_)_2_) relative to other 1000 ppm of testing solutions of interference ions as well as pure ethanol and deionized water.

In order to verify the selectivity of the sensor, 1000 ppm testing solutions of other ions which can cause possible interference were prepared. We applied 5 μL of each test solution to individual sensors for measuring at room temperature (27 °C). As shown in Figure 4, the signal responses of the PEDOT:PSS-modified nanomechanical sensors demonstrated high selectivity toward Pb^2+^ over other ions. The responses of relative resistance change for most interfering ions were lower than 0.001, which is less than the response obtained from a 10 ppm of Pb^2+^ solution. Furthermore, for the application in real samples, the concentrations of interfering ions are generally below 1000 ppm, which should not significantly affect the sensor response. Therefore, the sensor selectivity can be distinguished.

### 3.4. Reusability of the Sensor

**Figure 5:**
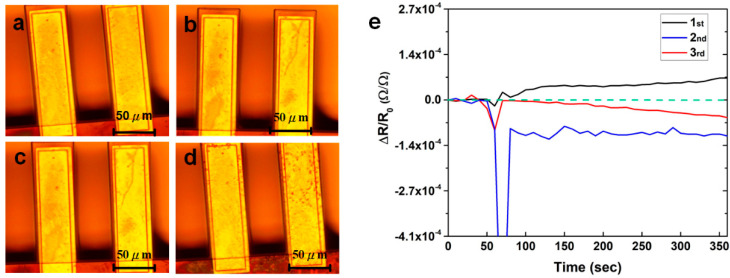
The optical microscopic images of PEDOT:PSS films coated on microcantilevers: (**a**) before exposure to 10 ppm of Pb^2+^ solution; (**b**) after exposure to 10 ppm of Pb^2+^ solution for the first measurement; (**c**) after the second measurement; (**d**) after the third measurement; and (**e**) each signal response of the sensor for the three separate measurements in a 10 ppm of pb^2+^ solution.

To study the reusability of the PEDOT:PSS-modified nanomechanical sensor, the relative resistance changes of the sensor were repeatedly measured three times in 10 ppm of lead ion solutions. As shown in Figure 5a–d, the optical microscopic images of PEDOT:PSS films coated onto microcantilevers appeared to flake after reacting with lead ions. Additionally, the more measurements that were taken, the more apparent flaking from the surface of the microcantilever happened. This may be explained due to the lead ions overoxidizing the PEDOT:PSS layers [25]. From the signal responses of the sensor, as shown in Figure 5e, only the first measurement displays the corresponding signal to the 10 ppm of Pb^2+^ solution. In the second and third measurements, signal responses are gradually reduced. Therefore, the proposed sensor is suggested for one-time measurement. However, due to the advantage of the standard CMOS MEMS process, the cost of single sensing chip is approximately estimated as $0.4. The sensing chip itself can be regarded as a disposable part of the device.

The comparison between this work and different reported Pb^2+^ sensing methods is summarized in Table S1. Most newly micromachined based Pb^2+^ sensors claim that they can achieve high sensitivity, good selectivity and relative fast response time. However, some methods incur high fabrication cost, and need sizeable measuring equipment and professional operators, which may not meet the timely, portable, low-cost needs of water quality testing. The proposed Pb^2+^ sensor in this work is not only provided with characteristics of high sensitivity, favorable selectivity and quick response time, but also has the ability to offer mass production and high-yield devices due to the contribution of standard CMOS manufacturing processes. The miniature integrated electrical readout and thermal effect self-elimination modus also make the sensor applicable for portable and on-site Pb^2+^ detection. In addition, the test sample does not need to be processed first, and the measurement process is quite simple. However, this method needs further examinations with real samples and comparison with the tested results with gold standard method.

## 4. Conclusions

In summary, a portable, real-time, cost-effective CMOS MEMS-based nanomechanical sensing array using the PEDOT:PSS modification layer, has been demonstrated for the detection of Pb^2+^. The detection is obtained by measuring the signal of relative resistance change resulting from the Pb^2+^ induced surface stress change of microcantilevers. The method can detect Pb^2+^ in an aqueous solution with the detection limit as low as 5 ppb; it can also show selectivity against other metal ions. The required sample volume for one measurement is only 5 μL. The sensing chips are fabricated by using the standard micromachining process integrated with an on-chip thermal effect elimination approach to achieve the purposes of mass production and portability. However, while the sensing array device is considered for single use, it can possibly prove reusable after redepositing the sensing layer. The proposed sensing platform shows its potential and feasibility for on-site sensitive water quality analysis and pollution monitoring.

## Supplementary Materials

The following are available online at https://www.mdpi.com/2079-4991/10/12/2454/s1, Figure S1: The response signals in the resistance change of the sensor before and after applying the real-time thermal effect eliminating method without other temperature control equipment, Figure S2: The schematic plot of PEDOT:PSS coating process, Figure S3: The plot of the measuring result of displacement is related to the resistance change of microcantilever structure, Table S1: Comparison of analytical performance by different reported methods measuring lead ions.
