# Supplementary material for: Portable Real-Time Detection of Pb(II) Using a CMOS MEMS-Based Nanomechanical Sensing Array Modified with PEDOT:PSS
Source: Nanomaterials (Basel). 2020 Dec 8;10(12):2454. doi: 10.3390/nano10122454 (PMC7763931; doi:10.3390/nano10122454)
Supplement: Supplementary file 1 [file nanomaterials-10-02454-s001.pdf]

Supplementary Materials

Portable real-time detection of Pb(II) Using a CMOS MEMS-based Nanomechanical Sensing Array Modified with PEDOT:PSS

Yi-Kuang Yen [1,2,*] and Chao-Yu Lai [1]

[1] Department of Mechanical Engineering, National Taipei University of Technology, Taipei 106, Taiwan; ykyen@ntut.edu.tw; t106568056@ntut.edu.tw

[2] Institute of Mechatronic Engineering, National Taipei University of Technology, Taipei 106, Taiwan; ykyen@ntut.edu.tw

[*]Correspondence: ykyen@ntut.edu.tw

S1. Thermal effect self-elimination method [1]

To apply the thermal effect self-elimination method, the sensing chip first needed to perform temperature calibration process to obtain the temperature functions in terms of the resistance changes of microcantilever and the embedded on-chip temperature sensor. The microcantilever sensor chip was adhered to the temperature control platform with thermal paste. Temperature gradient measurements were determined by using a multi-function digital meter with a division of labor and matrix relay. The temperature control setting was usually between 19 °C and 28 °C, with an interval of 1 °C, and each temperature lasted for 15 minutes. This temperature range was slightly adjusted depending on the ambient temperature during the experiment. When the temperature control platform started to operate, the multi-function digital meter will record the resistance signal of the on-chip temperature sensor and the microcantilever sensor at an interval of one data point every 10 seconds. When the temperature-to-resistance change was obtained, temperature functions of the on-chip temperature sensor and the microcantilever sensor can be established as following equations:

$$R_{Metal} = a_1(\mathrm{T}-\mathrm{T}_0)^2 + b_1(\mathrm{T}-\mathrm{T}_0) + c_1 \qquad \text{(S-1)}$$

$$R_{MCL} = a_2(\mathrm{T}-\mathrm{T}_0)^2 + b_2(\mathrm{T}-\mathrm{T}_0) + c_2 \quad \text{(S-2)}$$

During the measurement, the resistance value of the on-chip temperature sensor was brought into the formula (S-1) to reverse the real-time temperature of the micro cantilever sensor chip, and then the real-time temperature of the chip was brought into the formula (S-2) to calculate the resistance value of the microcantilever. The thermal effect of the ambient temperature on the microcantilever sensing chip can be corrected to obtain real signals in the measurement. The thermal effect self-elimination equation is as follows:

$$R_{real} = R_{measure} - R_{Thermal} \quad \text{(S-3)}$$

where $R_{measure}$ is the resistance value measured by the digital meter, and R_{real} is the real signal after the microcantilever sensing chip is thermally compensated.

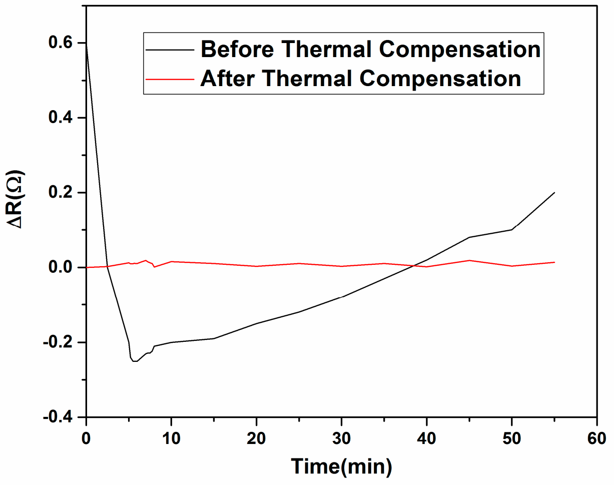

Figure S1. The response signals in the resistance change of the CMOS MEMS nanomechanical sensing arrays before and after applying the real-time thermal effect eliminating method without other temperature control equipment.

S2. Ink extrusion printing method

The coating process of PEDOT:PSS film used ink extrusion printing method is shown in Figure S2. The coated dry film thickness (t) can be controlled and calculated from the pump rate (Q), solid content (wt) ,the substrate motion speed (U), and the coating width (W), as shown below. In this work, the thickness can be calculated as 1.8 μm.

$t = (Q * wt) / (W * U) =(20/3600 \text{ mm3/s} *1.3\%) / (0.04 \text{ mm} * 1 \text{ mm/s}) = 0.0018 \text{ mm} = 1.8\ \mu m$

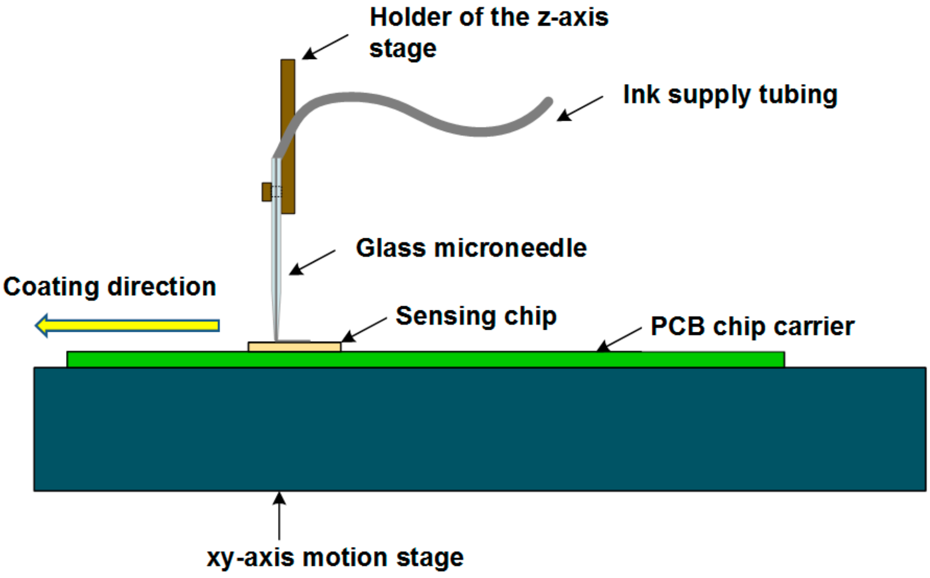

Figure S2. The schematic plot of PEDOT:PSS coating process

S3. Thermal effect self-elimination method [1]

In order to calculated gauge factor of the nanomechanical sensor, the ratio of displacement to resistance change of microcantilever should be obtained. The measurement was performed by controlling the amount of displacement (Δz) of the probe at the end of the microcantilever and simultaneously measuring the amount of

resistance change (ΔR) to obtain their ratio ($\Delta R/\Delta z$). The experimental result of the displacement (Δz) and resistance change (ΔR) of the nanomechanical sensor was shown in Figure S3. It can be obtained from the experimental results that $\Delta R/\Delta z = 5.4483\ (\Omega/\mu m)$ and the reference resistance R_0 of the microcantilever at 25 ° C is 7.23 (kΩ).

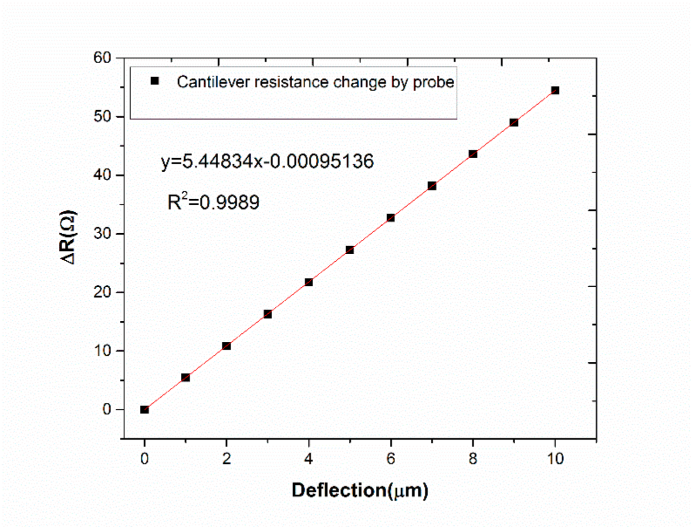

Figure S3. The plot of the measuring result of displacement is related to the resistance change of microcantilever structure.

Table S1. Comparison of analytical performance by different reported methods measuring lead ions.

Methods	LOD	Linear Range	Characteristics	Reference
DNAzyme-based QCM-D [1]	14 nM	46-3000 nM	High sensitivity, high selectivity and on-line detection/ High cost, slow response time	[2]
rGO/GSH-AuNP modified FET [2]	10 nM	0.1 ppb-0.011ppm	Fast response time, high sensitivity, high selectivity and user-friendly/ Non-standard and complicated modification process	[3]
DNAzyme-modified microcantilever sensor	10 nM	0.1 ppb-10 ppm	Low cost, high sensitivity, high selectivity and reusable sensor/ Non-portable platform	[4]

Hydrogel Swelling Microcantilever Sensor	1 µM	-	Low cost, good sensitivity/ Non-portable platform	[5]
Electrochemical Sensor Based on PYTS-CNT	0.02 µg/L	1 -100 µg/L	Fast response time and high sensitivity/ Interfering ions competing problem	[6]
PEDOT:PSS-modified CMOS MEMS piezoresistive microcantilever (This work)	5 ppb (24 nM)	10 ppb - 1000 ppm (48 nM - 4800 µM)	Low cost, high yield, high sensitivity, high selectivity portable platform and fast response time/ Not reusable	

[1] QCM-D: quartz crystal microbalance with dissipation monitoring; [2] FET: field-effect transistor

Reference:

1. Ku, Y.-F., Huang, L.-S. &Yen, Y.-K. A real-time thermal self-elimination method for static mode operated freestanding piezoresistive microcantilever-based biosensors. Biosensors 8, (2018).
2. Teh, H.B.; Li, H.; Li, S.F.Y. Highly sensitive and selective detection of Pb 2+ ions using a novel and simple DNAzyme-based quartz crystal microbalance with dissipation biosensor. Analyst 2014, 139, 5170–5175.
3. Zhou, G.; Chang, J.; Cui, S.; Pu, H.; Wen, Z.; Chen, J. Real-time, selective detection of Pb2+ in water using a reduced graphene oxide/gold nanoparticle field-effect transistor device. ACS Appl. Mater. Interfaces 2014, 6, 19235–19241.
4. Peng, R.-P.; Xing, L.-B.; Wang, X.-J.; Wu, C.; Chen, B.; Ji, H.-F.; Wu, L.-Z.; Tung, C.-H. Detection of Pb2+ in Aqueous Solution by Using a DNA-modified Microcantilever. Anal. Sci. 2016, 32, 1065–1069.
5. Liu, K.; Ji, H.-F. Detection of Pb2+ using a hydrogel swelling microcantilever sensor. Anal. Sci. 2004, 20, 9–11.
6. Jiang, R.; Liu, N.; Gao, S.; Mamat, X.; Su, Y.; Wagberg, T.; Li, Y.; Hu, X.; Hu,

G. A facile electrochemical sensor based on PyTS–CNTs for simultaneous determination of cadmium and lead ions. Sensors 2018, 18, 1567.
